# Challenges and Opportunities in Recruiting Research Participants Using Facebook: Lessons Learned from an Exemplar Study

**DOI:** 10.1177/08445621231207546

**Published:** 2023-10-18

**Authors:** Sarah Ashfield, Lorie Donelle, Maxwell Smith, Ève Dubé, Panagiota Tryphonopoulos

**Affiliations:** 1 6221 University of Western Ontario, London, Ontario, Canada; 2 Professor and Emily Myrtle Smith Endowed Professor of Nursing, College of Nursing, University of South Carolina, Columbia, SC, USA; 3 4440 Université Laval, 54470 Institut National de Santé Publique du Québec, Quebec, Canada

**Keywords:** Social media, recruitment, Facebook, methods

## Abstract

**Background:**

Facebook is a prominent social medial platform frequently used for business marketing. Researchers are starting to recognize the utility of this platform for developing research awareness, information dissemination, and more recently participant recruitment.

**Purpose:**

This paper will provide an overview of methods used in Facebook recruitment through an exemplar study. It will highlight successes and challenges and provide insight into future opportunities for its’ use.

**Methods:**

Two methods of Facebook recruitment are outlined in this paper: the use of Facebook groups and paid advertising. A step-by-step guide highlights how researchers can implement these specific methods of Facebook recruitment.

**Results:**

Facebook was successfully utilized to recruit participants in the exemplar study. Recruitment was completed over a period of 82 days with a total cost of $157.09 Canadian dollars.

**Conclusion:**

Facebook is a viable method of recruiting research participants. This method can be cost-effective, timely, and efficient in comparison to traditional research recruitment methods. However, one must balance the benefits and challenges of this type of recruitment.

## Background and purpose

Facebook is a prominent social media platform with over 1.98 billion daily users (Meta, 2022e). Social media platforms, including Facebook are frequently used by businesses for digital marketing and sales. The use of social media platforms has become a mainstream marketing strategy for business, spreading into marketing education, with many post-secondary institutions developing marketing programs dedicated exclusively to social media (Five Channels, 2007; Ontario College Application Centre, 2022).

Researchers have also begun recognizing social media platforms as valuable instruments to generate research awareness and support participant recruitment for research (Akard et al., 2015; Carter-Harris, 2016; Chu & Snider, 2013). Social media provides an opportunity to reach large numbers of participants, individuals that are geographically distant from the researcher, and those who may otherwise not be aware of research opportunities. Researchers have successfully utilized social media for participant recruitment in qualitative and quantitative research studies (Tustin et al., 2017; Whitaker et al., 2017). However, the potential to reach large numbers of individuals through social media platforms should be balanced with ensuring a representative sample with diverse participants through a comprehensive recruitment plan (Arigo et al., 2018). Representation of racial and ethnic minorities in research is important for many reasons, including the generalization of findings to highly diverse populations (George et al., 2014; Government of Ontario, 2022; Kamp et al., 2019). Hence, there is need for further guidance on methodological approaches to optimize social media platforms as recruitment tools (Arigo et al., 2018). This paper will provide an overview of methods for social media recruitment utilizing Facebook, with an exemplar study investigating parental vaccine decision making surrounding routine childhood and COVID-19 vaccines. The challenges and successes of Facebook recruitment will be addressed and suggestions for future research are highlighted.

### Background

The evolving COVID-19 pandemic created challenges for face-to-face research recruitment (Skeens et al., 2022). Non-pharmaceutical public health restrictions such as physical distancing and stay-at-home orders contributed to limiting in-person interactions, creating possible barriers between researchers and participants. Additionally, digital transformation propelled by the pandemic has changed the way that individuals interact and has facilitated online and hybrid forms of workplaces that include working from home, with an emphasis on online communication strategies such videotelephone software such as Zoom (Iivari et al., 2020; Trenerry et al., 2021). The use of social media platforms for creating, sharing and communicating online content continue to be prevalent methods of communication worldwide including within Canada (Pew Research Center, 2023; Statistics Canada, 2021).

Facebook remains a prevalent social media platform among Canadian users (Meta, 2022e; Statistics Canada, 2021). It is one of several social media platforms – Instagram, Messenger, WhatsApp- owned by the media corporation Meta; allowing for advertisements developed on one platform to be easily shared or used on other Meta social media platforms (Meta, 2022f). While the exact number of Canadian Facebook users is difficult to verify, in 2018, 9 out of 10 Canadians aged 15–34 years, and 8 out of 10 Canadians aged 35 to 49 years used social media on a regular basis (Statistics Canada, 2021).

Traditional recruitment methods in health sciences research (when there is no access to patient or population based contact information) include information posters, handouts, flyers, telephone calls, radio and print advertisements, mailing of letters, in-person information distribution, and, more recently, emailing of information (Patel et al., 2003). These methods can be time consuming, require up front financial commitment to develop and produce print materials, and use significant personnel to administer (Hoddinott & Bass, 1986). Researchers have identified that social media is a valuable tool that can be leveraged in several ways, including dissemination of research findings, administration of interventions, and now as a way to recruit individuals to participate in the research itself (Alotaibi et al., 2021; Brownson et al., 2018; Whitaker et al., 2017). Facebook has successfully been used as a method of recruiting participants in various health research areas, including smoking (Carter-Harris et al., 2016), violence against youth (Chu & Snider, 2013), among men who have sex with men (Hernandez-Romieu et al., 2014) and the impact of the pandemic on families (Skeens et al., 2022). Few studies have used Facebook recruitment to reach parents of young children (Skeens et al., 2022; Tustin et al., 2017). Researchers within various countries including Canada, Australia, United States, Germany, and Japan have employed Facebook recruitment with success (Whitaker et al., 2017). While the use of social media as a recruitment strategy in health sciences is a contemporary approach, it has demonstrated comparability or even advantages in accessing harder-to-reach and marginalized populations when compared to traditional methods (Akard et al., 2015; Hernandez-Romieu et al., 2014; Whitaker et al., 2017).

The social media platform Facebook was chosen as the primary recruitment method for a mixed methods research study we conducted, which investigated parental vaccine decision making. This approach was taken for several reasons. Firstly, Facebook is a digital environment wherein potential participants gather to look for vaccine information (Ashfield & Donelle, 2020; Brunson, 2013; Getman et al., 2018). Secondly, this research study was planned and conducted during the COVID-19 pandemic, with Ontario public health measures fluctuating and favoring physical distancing. Thirdly, existing evidence supports targeted social media recruitment as an efficient and cost-effective method of accessing parents for vaccine research, including vaccine-hesitant parents who may be harder to recruit using traditional approaches (Tustin et al., 2017). Lastly, social media recruitment is recognized as an ethical approach for recruitment of participants by the tri-council of Canada (Canadian Institutes of Health Research et al., 2022).

## Methods and procedures

### Participant recruitment using Facebook

The aim of this paper is to provide an overview on how Facebook recruitment was conducted, providing researchers with a step-by-step guide. This paper will also highlight the strengths and challenges of using Facebook as a recruitment method. The participant recruitment strategy using social media for our study contained two primary methods: the use of Facebook groups, and Facebook advertising. Prior to beginning any recruitment through Facebook, several steps were required to set up a Facebook account as this is required to fully utilize the platform's features. Ethics approval was obtained through the researcher's university research ethics board for the exemplar study.

Facebook groups are digital spaces where individuals with shared interests can gather to create virtual communities. These groups are either public (open to any Facebook user) or private (require permission from group administrators to join). Facebook advertising is generally used to create brand awareness surrounding commercial services and products (Lu, 2015). Used in this way, Facebook advertisements are targeted to specific audiences and future customers through various ad campaigns. Facebook advertisements have evolved from simple banners and images placed on the Facebook feed to include detailed interactive videos with specific marketing goals such as website visits, direct product purchases or sales conversions (Meta, 2022f). In our case, Facebook advertising was leveraged to create awareness about the opportunity to participate in a particular research study. This social media recruitment approach was informed by a Canadian study that recruited parents for a vaccine study exclusively through Facebook and from a scoping review that identified that parents who are making vaccine decisions and are present online, particularly on social media platforms (Ashfield & Donelle, 2020; Tustin et al., 2017). The steps below outline the process utilized to develop the Facebook recruitment strategy.

#### Facebook groups

Step one: Prior to beginning the recruitment process, a Facebook profile and page were created specifically for the study, using the same process that an individual would use to create a personal account. A business account is not required to run an advertising campaign on Facebook; however, a page is required. This page can be linked with any Facebook account (personal, business or a new account only utilized to run a research recruitment campaign, as done here). A Facebook page is required to run ads within the Facebook platform; pages are a free way for businesses, brands, celebrities, causes, and organizations to reach audiences (Meta, 2022a). There are six main categories of Facebook pages, including local business or place, community organization or institution, brand or product, artists band or public figure, entertainment, and cause or community (Meta, 2022a). These categories are rapidly changing with the ongoing addition of page subcategories and an updated pages experience in the fall of 2022 (Meta, 2022b).

Step two: Once an active Facebook account and page are established, researchers can explore and identify Facebook groups related to the stated research purpose to target participant recruitment efforts. In our case, a search for various parenting interest groups within Ontario was performed as the targeted participants were parents living in one specific Canadian province.

Once logged into the Facebook platform, a search box can be utilized for locating and identifying Facebook groups. The use of search terms that identify groups where potential participants may gather can be entered into the search box to find Facebook groups that the researcher can request to join. Something to consider when searching for Facebook groups, is the wide variety of names or descriptors individuals may use to label or identify themselves when creating their Facebook group. Including cities and communities from across the desired geographical area, varied community sizes and compositions, and inclusion of minority populations are some of the factors that one may consider when using search terms to locate Facebook groups where participants are present. An example of descriptor words utilized in the exemplar include: Montessori, moms, Waldorf, neighbourhood, parents group, homeschool, Muslim, nannies, Indigenous. To enhance the diversity of potential research participants, search for Facebook groups using the desired demographics (eg., specific religious, ethnic, or cultural group) of targeted participants as demographic diversity of participants recruited online can be a challenge (Bonevski et al., 2014).

Step Three: For this particular study, once a potential group was identified if the group was public, it was joined; if the group was private, a request was made to join the group. A total of forty-three groups were joined to support participant recruitment for the study on parents’ vaccine decision-making.

Step Four: Once a group was joined, one of several methods were utilized to gain permission to place a post within the group. If advertisements were permitted, the recruitment poster and a direct link to the survey were posted to the group's newsfeed. If there were no specific instructions about personal posts or advertisements, a request was sent to a group administrator for permission to post the recruitment poster onto the group's newsfeed.

Step Five: Once permission was granted, the recruitment poster was posted on the targeted (parenting) groups’ newsfeed. As a measure to maintain participant confidentiality, all recruitment posters placed within groups had the ‘commenting’ function disabled. To further ensure participant confidentiality, a statement was posted on the study's Facebook profile stating, “This Facebook page has been created as part of [study name], it is conducted by [ name(s) of researcher(s)] through the University of the principal investigator. Note: a ‘like’ or ‘follow’ or sharing this post could indicate that you are publicly identified with this study.” These additional measures were taken as a precaution to educate the potential participants about how their social media activities could potentially link them to the research study.

#### Facebook advertising

**Figure 1.**
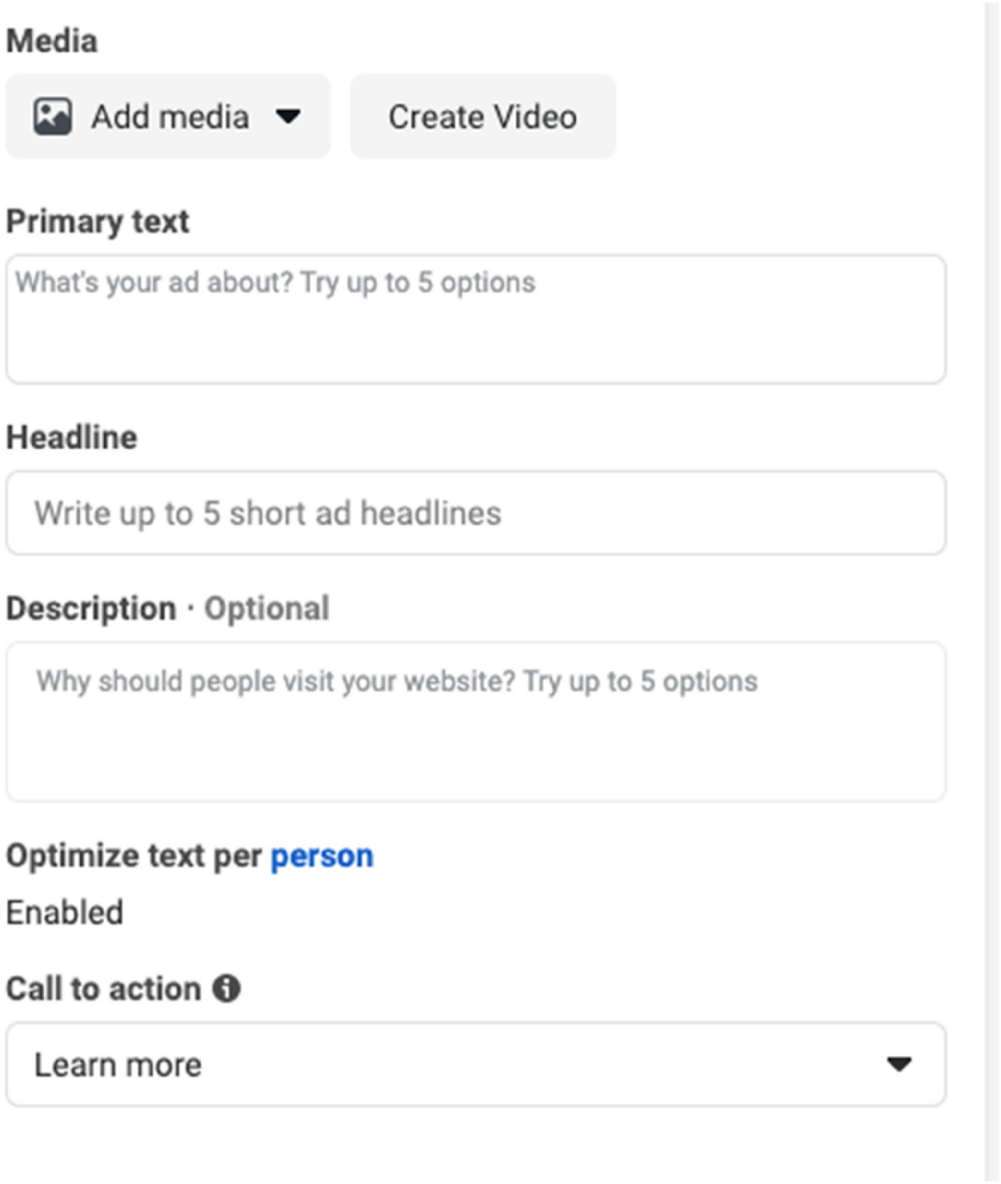
Facebook advertisement template.

The second participant recruitment method we utilized was the use of Facebook advertisements. An advertisement was created from the research Facebook page using a single image format. There are several advertisement formats within Facebook to choose from, including single images, videos, and multiple images. Facebook has a set format for single image advertisements as pictured in Figure 1.

**Figure 2.**
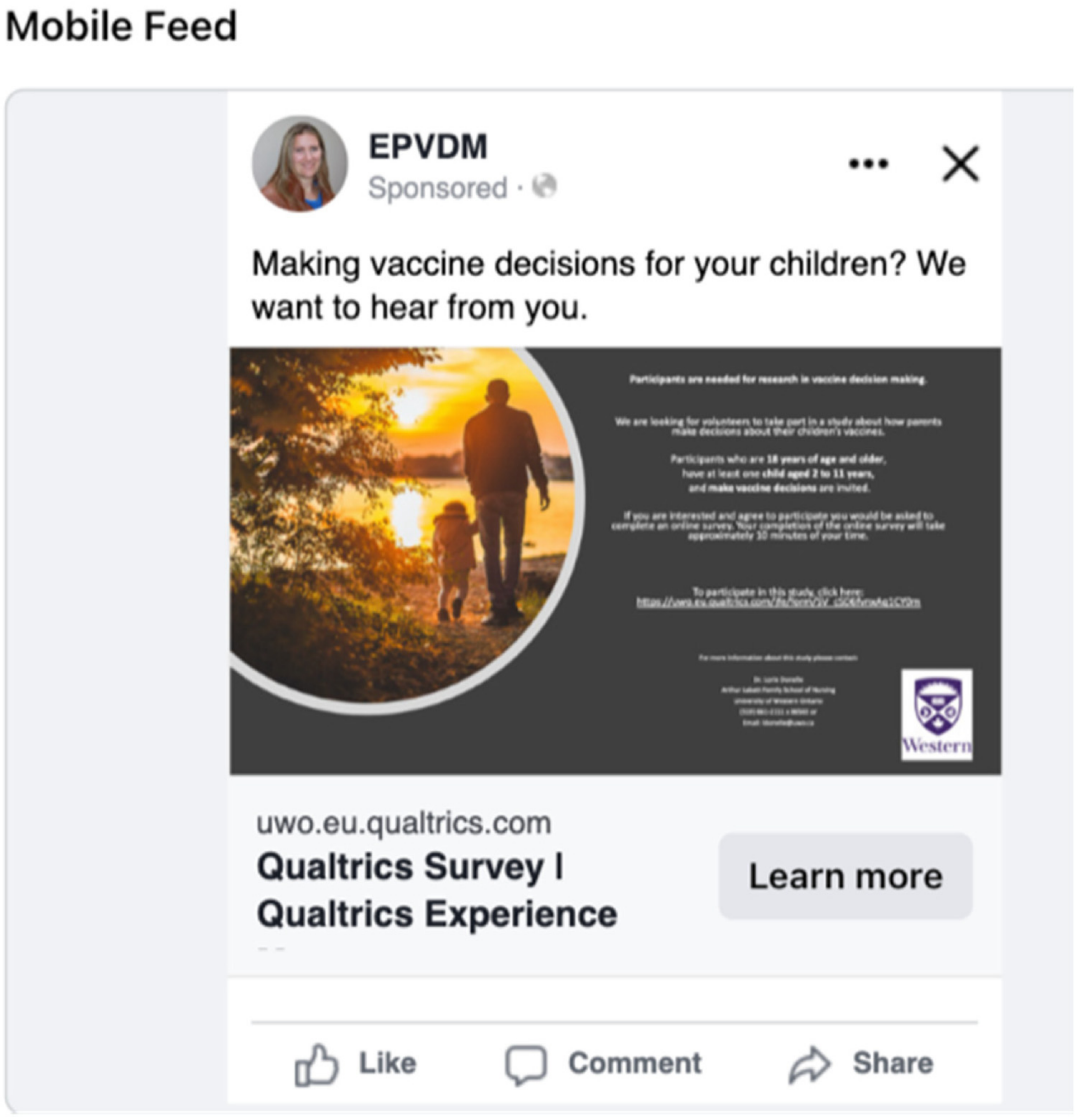
Exemplar study facebook advertisement example.

Step One: Setting the main portions of the single image advertisement requires three pieces of information: a description, an image, and a call-to-action label. The description appears at the top of the add and asks the user to describe what is being promoted. The wording “Making vaccine decisions for your children? We want to hear from you” was utilized, the image was the approved recruitment poster, and the *call-to-action* button selected was “Learn more”. Facebook advertising allows one to choose only from pre-determined *call-to-action* wording. When individuals clicked on the *call-to-action* button, they were sent to the study survey directly. Figure 2 shows a mobile view of the completed Facebook advertisement that was used in our study ad campaign.

Step two: Setting an objective for the campaign is required by Facebook. This allows for monitoring the success of the advertisement. The objective of the campaign was set to ‘conversion’, which refers to having people take an action as a result of the advertisement (Meta, 2022c). The conversion goal of this advertising campaign was to have individuals complete an online survey. Traditional commerce advertising conversion goals typically aim to drive website traffic or purchase specific products. Conversion advertisement campaigns through Facebook require a website with a pixel link (Meta, 2022c). A pixel is a digital method of tracking results from the advertisement to the targeted action (Meta, 2022c).

Step three: The pixel link must then be activated. A Google site was created, and a pixel was generated through the Facebook advertisement setup and embedded within the Google site. This was a simple website that had basic recruitment information with a link to the survey. The sole purpose of this step is to meet the requirements of the Facebook advertising conversion objective; the advertisement did not drive users to this website. Without having a pixel embedded in an external website, Facebook will not run an advertisement campaign.

Step four: Once the conversion goal and pixel were completed an audience was determined. The general audience description was set as gender all, adults 18–50 years of age, English language, and the geographical area of Ontario, Canada. A specific targeted audience, while not mandatory, was selected to target advertisements to individuals most likely to meet the study's inclusion criteria. Pre-determined groups that best fit the study's audience were chosen, including: “parents all”, “parents with early school aged children 6–8 years”, “parents with pre-teens 9–12 years”, and “parents with pre-schoolers 3–5 years.”

Step five: Placement of the advertisement must then be selected. This allows researchers to either let Facebook automatically place the ad on Facebook stories and feeds or limit locations. This setting was set to ‘automatic’ allowing the algorithm to determine advertisement dissemination targets. Different formats may appear better in various locations; for example, a video may best appear on stories as opposed to a fixed image such as the one utilized in this campaign.

Step six: Provide a budget. Initially, a limited daily budget of $3.00 per day was selected for an advertisement trial without spending a large sum of money. Once the campaign demonstrated survey completion, the budget was increased to $7.00 per day for the duration of the campaign.

Lastly after all of the above six steps are completed, the campaign must be submitted for approval to Facebook, in the exemplar case, approval took less than a day (Meta, 2022c). The advertisement starts running once the approval is completed by Facebook.

## Results

Recruitment for our study was completed over 82 days, with a total of 539 link clicks and a total of 231 completed surveys. The reach of this campaign was 25,407 people. A total of 43 Facebook groups were joined at a cost of $0, the entirety of the cost was for Facebook paid advertising. The total cost of recruitment for this mixed methods study was $157.09 Canadian dollars, at a cost of $0.68 per participant.

Of the 231 participants, 12.1% (N = 28) were male, 84.4% (N = 195) were female, 0.9% (N = 2) were non-binary/third gender, and 2.6% (N = 6) preferred not to report their gender identity. There were no participants under the age of 25 years, 4.3% (N = 10) were 26–30 years, 56.3% (N = 130) 31–40 years, and 39.4% (N = 91) 41 years of age and above. Most participants had one or two children aged 2–11 years of age living in their household with 42.2% (N = 98) having one child, 40.7% (N = 94) having two children, 12.6% (N = 29) having 3 children and 3.9% (N = 9) having 4 or more children.

## Discussion

Facebook was used as an effective, timely, and economical method of recruiting participants for research. The exemplar study outlined in this paper demonstrated successful use of Facebook through recruitment of over 200 participants in 82 days.

### Opportunities

Social media is effective and time efficient in comparison to traditional recruitment methods such as posters, in-person information sessions, phone calls, newspapers or flyer distribution (Reagan et al., 2019). Traditional recruitment methods may take longer than social media methods due to time consuming administrative factors such as response time from completion of survey, transit time for mailed information, and printing time. Social media recruitment eliminates these administrative tasks.

Another benefit of Facebook recruitment is the ability to reach participants who prefer anonymity and wish to protect their privacy. Some subpopulations may not be comfortable identifying themselves, may not be physically present in a specific location, or wish to remain anonymous. In the exemplar study, additional measures beyond what was required by the research ethics board were utilized to ensure privacy. A statement identifying that ‘following’ or ‘liking’ the page may indicate affiliation with the study; commenting was disabled in all Facebook group feed posts. However, individuals can comment on paid Facebook advertisements a feature that is not controlled by individuals who create the ads. Despite these privacy challenges Facebook has successfully been used as an effective method of recruiting historically stigmatized populations, including men who have sex with men, persons who identify as LGBT2Q+, and cannabis users (Barratt et al., 2015; Brodar et al., 2016; Hernandez-Romieu et al., 2014; Vial et al., 2014).

Facebook provides a cost-effective method of recruiting and when compared to traditional methods of recruitment is less expensive (Wasfi et al., 2021). This study cost less per participant ($0.68) compared to other studies that also utilized Facebook to recruit participants (Barratt et al., 2015; Juraschek et al., 2018; Skeens et al., 2022; Weiner et al., 2017; Whitaker et al., 2017). A range of costs per participant is present in the literature from $1.18 per participant in Weiner et al.'s study (2017) to a high of $436 per participant (Juraschek et al., 2018).

The presence of study participants’ online on Facebook platforms provides an opportunity to facilitate post-enrollment communication and knowledge mobilization. Publication of research findings in academic journals are one successful method of knowledge mobilization in the academic and research community, laypersons are unlikely to have access to these journals and lack the literacy to interpret the findings. However, posting research findings on Facebook or other social media platforms where specific populations are present provides an opportunity for dissemination of important research findings (Smith & Watson, 2016).

The use of Facebook advertising requires one-time approval for posting recruitment materials. Traditional recruitment methods necessitate permission to distribute recruitment materials at each organization or establishment, which not only have enhanced labour requirements but can also impose significant wait times for individual responses. This approval process expends researchers’ time and resources to obtain permission and communicate with each institution and organization. The use of Facebook eliminates these administrative tasks and the travel time to physically develop, produce, and distribute materials.

### Challenges

There are several challenges with using Facebook as a recruitment method. While there is a diversity of individuals present online, obtaining participant samples that include marginalized and disenfranchised individuals can be difficult (Bonevski et al., 2014). This was evident in the example study where the majority, 87.9% (N = 203) of participants were White, another 8.2% (N = 19) were of various other visible minorities, and 3.9% (N = 9) preferred not to respond. In 2016, 29.3% of Ontario's population was comprised of individuals who identified as members of a visible minority (Government of Ontario, 2022). Other researchers have also found that obtaining an ethnically diverse participant group through Facebook recruitment was challenging, particularly for studies that recruited parents (Akard et al., 2015; Skeens et al., 2022). For example, Skeens’ et al. (2022) American study included 90% White participants and targeted zip code recruitment in an attempt to increase the ethnic diversity of participants. Other studies that recruited participants via Facebook who weren’t parents also reported predominantly White participants (Bauermeister et al., 2012; Ramo & Prochaska, 2012; Ramo et al., 2014; Schwinn et al., 2017; Wasfi et al., 2021). Most disadvantaged individuals (eg., homeless or underhoused) will not likely be able to respond to the survey due to a lack of access to a cell phone or computer required for digital survey completion.

There were challenges joining Facebook parenting groups related to either group moderator restrictions or Facebook account limitations. Group moderators or administrators can place restrictions or requirements on those requesting to join their group that led to refusal or denied access. For several groups, membership was restricted to individuals with a Facebook profile that had existed for at least 3 months. Some Facebook groups require acknowledgment of a list of group rules and/or questions for acceptance into the group. When this occurred, the research rationale was introduced, and the purpose of recruiting participants was explained. At times this resulted in an automatic refusal of group membership. Facebook groups that are protected by moderators may reflect the groups desire for a shared community closed to outsiders. While challenging for researchers this sense of community and supported social network is one of the benefits that online closed groups provide their members. In addition to this challenge of joining closed groups, Facebook also limits the number of groups that can be joined at one time. While this number isn’t available through their website, after joining multiple groups in one day a notification that no further groups could be joined was received. To avoid this issue, only 3 to 5 groups joined per day is recommended.

One major limitation of Facebook advertising is that Meta utilizes automated review of advertising (Meta, 2022c). This can lead to restriction of one's advertising campaign inadvertently if it is considered to violate the commerce-focused advertising standards. This occurred in our study where Facebook restricted advertising privileges. The Facebook page associated with an account is used to run advertising. Initially, the page for this campaign was categorised as a business. However, after only four days of running the advertisement, the page was flagged, the account restricted, and the pages’ advertising privileges revoked, reportedly for not following Facebook's advertising standards. After submitting an appeal for both the advertisement and the page, Meta's final decision was to label our Facebook page as untrustworthy due to lack of transparency about what the page was ‘selling’ and declined to rescind the advertising restriction. Further inquiries through Meta support revealed that staff do not have access to the advertisement or page review process. It was also identified that Facebook administers an approval prior to adds running. However, there is limited clarity on how this is performed. Meta does acknowledge that Facebook advertisement approval is mainly an automated process (Meta, 2022c). To circumvent this advertising restriction, a new page was created with the category of “Education”. The same advertisement successfully ran under this new page classification without advertisement restriction. This reflects a challenge of using a business or sales developed advertising technique to recruit research participants.

Greater opportunity to enhance participant recruitment includes the use of multiple advertisement formats. While approval of one traditional research poster is the standard, engaging in discussion with the ethics review committee, on the benefits of multiple media formats for social media recruitment, may increase the likelihood of approval of other recruitment materials in various formats. Expanded placement within Facebook advertisements would be possible with the approval of a video or multiple images allowing for the creation of a reel or video advertisement. Social media and technology continue to evolve to allow researchers to reach participants that may be less accessible through traditional recruitment methods. Even though social media recruitment may reach some populations not traditionally reached in research recruitment, it will not reach those who do not or cannot use social media. The ethical issues of privacy and data protection require continued knowledge acquisition around social media and digital technologies. Increasing awareness and knowledge around how various social media platforms obtain and track personal information is important for researchers and individual social media users alike. Further to this, the distribution and sale of private information collected online via social media platforms is an area of emerging concern. Many confidentiality and privacy concerns are avoided when using social media to direct potential participants to digital surveys where no personal data is collected. Involvement of experts in the areas of social media and information privacy to assist researchers and institutional research ethics boards in keeping pace with these rapidly evolving areas is recommended.

## Conclusion

Facebook is a feasible method for research recruitment; researchers will need to balance the advantages of utilizing this social media platform against the challenges listed. Facebook group and paid advertisement recruitment can be economical and may be helpful for researchers, especially student researchers, with limited funding. Cost savings are generated from a reduced need for human personnel, elimination of travel time, and minimal to no administrative tasks which reduce overhead costs associated with traditional recruitment methods. Facebook advertising budgets can be closely monitored with daily limits, allowing for a trial of various advertisements prior to running a full campaign. Joining Facebook groups to place recruitment posters on newsfeed is of no cost. Facebook advertising requires minimal labour once an advertising campaign is in place.

However correct, development and placement of an advertising campaign requires knowledge of Facebooks advertising platform and campaigns can be hindered by Facebook restrictions. Consider familiarizing oneself with the Facebook advertising platform prior to placing research advertisements. Consultation with a social media marketing expert may be another strategy to familiarize oneself with Facebook advertising strategies; however, may increase the financial costs.

Flagging an advertisement as a social issue prior to submitting it to Meta for approval may reduce the chances of the campaign being restricted through Facebook (Campbell-Smith, 2022). This may be especially helpful if the research topic is of current political or social interest, as Facebook has algorithms that flag specific wording. However, this may have the potential to create selection bias as participants would likely see the advertisement flagged by Meta as a social issue. Correct labelling of the page associated with the Facebook profile is critical in avoiding Facebook restrictions. Algorithms and restrictions are constantly changing, so researchers need to be flexible and adapt to these changes quickly.

Consider creating and tailoring a separate Facebook page for each research project. The development of a new page for each project creates the challenge of having to establish a new group of contacts. However, if one is using paid advertising, this may not be an issue. Consider developing and maintaining a research Facebook account that can be used for a program of research. One advantage of this is that a Facebook profile that has ‘built up’ friends and interactions with other Facebook accounts increases the trust level of your account. Facebook uses not only your posts but also your interaction with others to verify the authenticity and trustworthiness of your account to ensure that you meet its community standards (Meta, 2022c). Consideration that maintaining an established Facebook account does allow Facebook to record and track your interactions and may have greater implications for privacy for the researcher as well as potential future and ongoing participants. For example, following a page makes ones Facebook profile visible to the page administrator as well as any Facebook user who looks at the ‘followers’ of a page. There is also an identified lack of transparency and disclosure around data sharing between smartphone apps and social media platforms, including Facebook specifically (Huckvale et al., 2019). This could potentially have participants and researchers unknowingly sharing health information contained in these applications with various social media platforms or web browsers (Huckvale et al., 2019; Nicholas et al., 2020).

Consider recruitment on social media platforms targeted to one's research. Meta is a parent company of other social media platforms which allows for easy one click access to potential participants who are known to be active on other social media platforms such as Instagram. However, this highlights some of the privacy concerns identified above and may lead to erosion of trust between researchers and participants. Participants of varying demographics may be more active on social media platforms that aren’t owned by Meta. Social media recruitment is restricted to active users, consider using other recruitment methods for targeting participants who are not present on social media.

Digital literacy may also be a limiting factor with this recruitment method. The sample of participants in this study demonstrated homogeneous ethnicity with 87.9% being White. Consideration of other methods of recruitment may be required to obtain a more heterogeneous sample of research participants. The messaging platform What's App may be a future area to explore in reaching ethnic minorities. Meta owns this platform and has recently started promoting it as a potential for advertising, allowing advertisements to easily transition across their platforms (Meta, 2022d).

Consider using the social media platform Facebook as a method of recruiting participants for research. While this method may not be suitable for all populations, it was a successful method of reaching parents living in Ontario in this mixed methods study.
